# A 3D MR-acquisition scheme for non-rigid bulk motion correction in simultaneous PET-MR

**DOI:** 10.1186/2197-7364-1-S1-A37

**Published:** 2014-07-29

**Authors:** Christoph Kolbitsch, Claudia Prieto, Charalampos Tsoumpas, Tobias Schaeffter

**Affiliations:** Division of Imaging Sciences and Biomedical Engineering, King’s College London, London, UK; Division of Medical Physics, University of Leeds, Leeds, UK

Positron emission tomography (PET) is commonly used to detect tumours and assess the progress of cancer treatment by measuring standardized uptake values (SUV). Physiological motion during data acquisition can negatively impair obtained SUV. Simultaneous PET-MR combines metabolic PET information with high-resolution anatomical MR images and has the potential to minimize motion artefacts.

A 3D MR-acquisition scheme is proposed which allows for the automatic detection and correction of non-rigid bulk motion shifts, i.e. changes of the patient’s position leading to non-rigid changes of the patient’s anatomy, in PET and MR images (both T1-weighted gradient echo and T2-weighted turbo spin echo images) without an increase in scan time.

Six respiratory gated T1- and T2-weighted 3D data sets with an isotropic resolution of 1.5mm were obtained using a radial phase encoding (RPE) acquisition. Volunteers are asked to move the abdomen during data acquisition resulting in overall 19 movements at arbitrary time points. RPE allows for a retrospective reconstruction of dynamic 3D MR images with different temporal resolutions from the same data. Non-rigid bulk motion is detected and corrected from these dynamic images. A simultaneous PET acquisition is simulated to assess the effect of motion correction on image quality and SUV for lesions with different diameters.

Every bulk motion shift was accurately detected and corrected. Non-rigid motion fields describing the different motion states were obtained with an accuracy of 1.71 ± 0.29mm. The PET simulation showed SUV errors of up to 67% due to bulk motion which could be reduced to 10% with the proposed motion compensated approach.

The presented MR acquisition scheme yields both high resolution 3D anatomical data and highly accurate non-rigid motion information without an increase in scan time. This method strongly improves both MR and PET image quality and ensures an accurate assessment of tracer uptake.

